# EMNet: A Novel Few-Shot Image Classification Model with Enhanced Self-Correlation Attention and Multi-Branch Joint Module

**DOI:** 10.3390/biomimetics10010016

**Published:** 2025-01-01

**Authors:** Fufang Li, Weixiang Zhang, Yi Shang

**Affiliations:** School of Computer Science and Cyber Engineering, Guangzhou University, Guangzhou 510006, China; zhangwx@e.gzhu.edu.cn (W.Z.); 2112206124@e.gzhu.edu.cn (Y.S.)

**Keywords:** few-shot image classification, few-shot learning, enhanced self-correlated attention, multi-branch joint module

## Abstract

In this research, inspired by the principles of biological visual attention mechanisms and swarm intelligence found in nature, we present an Enhanced Self-Correlation Attention and Multi-Branch Joint Module Network (EMNet), a novel model for few-shot image classification. Few-shot image classification aims to address the problem of image classification when data are limited. Traditional models require a large amount of labeled data for training, while few-shot learning trains models using only a small number of samples (just a few samples per class) to recognize new categories. EMNet shows its potential for bio-inspired algorithms in optimizing feature extraction and enhancing generalization capabilities. It features two key modules: Enhanced Self-Correlated Attention (ESCA) and Multi-Branch Joint Module (MBJ Module). EMNet tackles two main challenges in few-shot learning: how to make an effective important feature extraction and enhancement in images, and improving generalization to new categories. The ESCA module boosts the precision in extracting crucial local features, enhancing classification accuracy. The MBJ module focuses on shared features across images, emphasizing similarities within classes and subtle differences between them. This enhances model adaptability and generalization to new categories. Experimental results show that our model performs better than existing models in one-shot and five-shot tasks on mini-ImageNet, CUB-200, and CIFAR-FS datasets, which proves the proposed model to be an efficient end-to-end solution for few-shot image classification. In the five-way one-shot and five-way five-shot experiments on the CUB-200-2011 dataset, EMNet achieved classification accuracies that were 1.27 and 0.54 percentage points higher than those of RENet, respectively. In the five-way one-shot and five-way five-shot experiments on the miniImageNet dataset, EMNet’s classification accuracies were 0.02 and 0.48 percentage points higher than those of RENet, respectively. In the five-way one-shot and five-way five-shot experiments on the CIFAR-FS dataset, EMNet’s classification accuracies were 0.19 and 0.18 percentage points higher than those of RENet.

## 1. Introduction

In the realms of machine learning, the availability and richness of large datasets are crucial for training robust and efficient models. However, in many practical applications, especially in fields such as medical imaging and biometric recognition, acquiring extensive labeled data can be costly, impractical, or even impossible. Few-shot learning (FSL) emerges to address this challenge by learning from a minimal number of samples. Despite significant progress in FSL in recent years, effectively utilizing limited data to build an efficacious machine learning model remains an open question. FSL aims to mimic the human ability to learn impressively from a limited number of examples. Traditional metric learning approaches tackle the FSL problem through meta-learning deep embedding functions [1,2,3,4,5]. Facing scenarios with limited samples, most few-shot classification algorithms adopt a meta-learning-based training strategy to simulate human rapid learning capabilities and avoid model overfitting on training data. Under this training strategy, model training is divided into two phases: meta-training and meta-testing. During the meta-training phase, the model is trained on a dataset $D_{train}$ containing a rich set of base categories. The objective is to learn and optimize the model’s parameters through these known categories, enabling the model to capture the general features between categories. In the meta-testing phase, the model is evaluated using a new dataset $D_{test}$, which includes categories $C_{test}$ not seen during the training process. In both phases, the training adopts an N-way K-shot strategy, where, in each meta-task, K samples from N different categories are selected to form the support set, and the query set consists of additional samples. The goal of both the meta-training and meta-testing phases is to enhance the model’s recognition accuracy of new categories while maintaining robustness to known categories. Through this approach, few-shot learning models can not only learn to classify seen categories but also effectively recognize and differentiate new categories not encountered during training. This training mode helps maintain high adaptability and flexibility in the model amidst the real world’s changing environments. However, applying these embedding functions to new classes unseen during training poses a challenge due to the unique mechanism of meta-learning, often leading to overfitting on irrelevant features [6]. To combat overfitting, RENet [7] introduces relational embedding, focusing on the relationships between spaces. Our work not only emphasizes the spatial relationships but also pays close attention to intra-class similarities and captures subtle inter-class differences through attention mechanisms.

We introduce Enhanced Self-Correlation Attention (ESCA) and Multi-Branch Joint Module (MBJ Module).

ESCA extracts crucial local area features within images through enhanced computation and convolution operations, enhancing them via self-attention. MBJ filters out irrelevant features, analyzes shared features across images, and combines considerations of intra-class similarities and inter-class subtle differences to produce attention results from multiple branches, ultimately integrating all attention outcomes.

In the 5-way 1-shot and 5-way 5-shot experiments on the miniImageNet dataset, EMNet’s classification accuracies were 0.02 and 0.48 percentage points higher than those of RENet, respectively. In the five-way one-shot and five-way five-shot experiments on the CIFAR-FS dataset, EMNet’s classification accuracies were 0.19 and 0.18 percentage points higher than those of RENet. Experiments demonstrate that our EMNet, comprising the ESCA and MBJ modules, achieves end-to-end training, precise localization of key areas of target objects, increased feature recognition, and significantly improves the classification accuracy of few-shot images. This is proven in experiments on three standard datasets—CUB-200, CIFAR-FS and miniImageNet.

Section 2 will provide a detailed introduction to related work and background knowledge, including few-shot learning and existing methods. Section 3 will introduce the structure and principles of the EMNet model, including a detailed description of the ESCA and MBJ modules. Section 4 will briefly describe the training strategy of EMNet. Section 5 will present the experimental setup and results analysis, including the experimental results on the mini-ImageNet, CUB-200, and CIFAR-FS datasets. Finally, Section 6 will conclude the paper.

## 2. Literature Review

### 2.1. Few-Shot Image Classification

Currently, the principal approaches to few-shot image classification can broadly be categorized into meta-learning and transfer learning. The aim of few-shot classification is to rapidly adapt to new tasks based on only a few available samples. Herein, we summarize the pertinent literature for both learning strategies.

**Meta-learning.** Meta-learning was initially proposed in the 1990s but did not gain widespread use at the time [8]. It was not until the widespread application of deep learning that researchers began to employ meta-learning techniques to optimize deep learning models [9,10]. In meta-learning, datasets are generally divided into meta-tasks, with task-specific training employed to develop a set of learning rules. During training, the model learns how to make accurate predictions in scenarios of scarce samples. During testing, the model classifies new categories without updating.

Among the many branches of meta-learning, methods based on metric learning and those based on initialization are two prominent variants [11,12,13,14,15]. Metric-based strategies aim to construct a metric space where samples of the same category attract each other, while those of different categories repel. For instance, MatchingNet [11] introduces a framework for matching query and support samples across different tasks, employing an LSTM module to maintain context information of tasks. ProtoNet [12] further simplifies representation learning by calculating class centroids and using the cosine similarity between query samples and these prototypes for classification decisions. Additionally, RelationNet [13], as a representative method based on metric learning, introduces a CNN-based relational module focused on handling combinations between query samples and class prototypes, thus directly learning in the metric space. Jiang [16] et al., aiming to enhance RelationNet’s [13] feature extraction capability, proposed a CNN pyramid structure for multi-resolution feature generation, facilitating the capture of features from coarse to fine, thereby improving the model’s generalization ability for new categories. In our research, we adopt a metric-based learning approach. Unlike traditional metric-based methods, which typically process features in the support and query sets independently, our strategy focuses on exploring and utilizing the deep semantic connections between the support and query sets. This unique approach enables our model to focus more on key targets rather than mere surface feature matching.

The core goal of initialization-based methods is to find an optimal starting point for the model’s parameters, crucial for enabling the model to rapidly adapt to new tasks with minimal adjustments. In this field, Model-Agnostic Meta-Learning (MAML) [14] stands out as a landmark method. MAML’s [14] versatility allows it to be applicable to various models, focusing on finding network initialization settings that accelerate adaptability to new tasks. Specifically, such optimal initialization enables the model to show significant performance improvements after just a few steps of gradient descent when faced with new tasks. Latent Embedding Optimization (LEO) [15] emerged to address challenges MAML [14] faces in high-dimensional parameter spaces. By learning latent generative representations of model parameters, LEO [15] avoids direct optimization in high-dimensional spaces, thus enhancing the efficiency and quality of optimization. Further research, such as the work by Raghu et al. [17], explored the efficacy of MAML [14], revealing its advantages in feature reuse mechanisms, beyond its rapid learning capabilities. They also proposed a simplified MAML [14] learning strategy, updating parameters in the inner loop only in the network’s last layer, simplifying the training process and preventing overfitting. These improvements not only enhance the model’s adaptability to new tasks but also offer significant advantages in computational resources and time efficiency.

**Transfer learning.** In the traditional paradigm of transfer learning, the network is first pre-trained on a benchmark dataset with a rich variety of categories, followed by a fine-tuning process to adapt the network to new categories [18,19,20]. When deep neural networks are used as feature extractors, this method can demonstrate significant generalization performance. However, researchers such as Chen et al. [18]. emphasize that to avoid overfitting, it is crucial to keep the weights of the feature extractor unchanged.

### 2.2. Attention Mechanisms

Attention mechanisms were initially proposed in neural network translation models, aiming to extract more significant discriminative features by emphasizing key local areas [21]. This mechanism has achieved significant breakthroughs in both computer vision and natural language processing fields. Taking the transformer model introduced by Ashish Vaswani et al. [22] in 2017 as an example, it marked the beginning of a new era in the development of large models, where the self-attention mechanism played a crucial role. Further, SENet [23] introduced a channel attention module specifically designed for image classification, significantly enhancing the network’s overall representational capability. Building on this, ECANet [24] further optimized the attention mechanism by proposing an efficient channel attention module that does not require dimensionality reduction, enabling effective cross-channel interactions. Continuing this trend, CBAM [25] integrated features of SENet and ECANet, adding a spatial attention module after channel attention, forming a comprehensive dual attention mechanism that effectively emphasizes key features in both spatial and channel dimensions of images. Despite these modules showing certain limitations in handling smaller datasets, mainly due to their excessive reliance on the prior knowledge of base categories and poor performance on entirely new category images, methods based on neighborhood relevance [26,27] can compute image self-correlation but often at the cost of high computational load and increased model complexity. Quang Huy Nguyen et al. [28] proposed the cosine attention mechanism, enhancing the representation and relevance analysis of support set and query samples, thereby obtaining more stable and accurate results in few-shot tasks. Contrasting previous work, MBJ transforms and refines input features, effectively preserving key information while delving into the intrinsic structural patterns of the data. MBJ enhances model reliability through two parallel paths. Firstly, one path employs four-dimensional convolution techniques to meticulously assess the cosine similarity between features, effectively filtering out irrelevant information. Then, the other path focuses on mining intra-class similarities and inter-class nuances, providing a deeper understanding of the data. Ultimately, integrating the logical outcomes of both paths improves the accuracy of few-shot image classification.

## 3. Method

### 3.1. Overview

**Figure 1:**
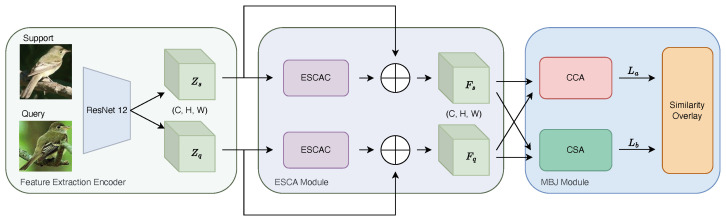
The overview of our proposed EMNet. We use ResNet 12 as a feature extractor. After that, the ESCA module enhances these base features via self-enhanced correlational attention. Finally, MBJ constructs a reliable similarity matrix and provides a result for the classification task.

In this subsection we will introduce the structure of our proposed EMNet. The overview situation of EMNet is illustrated in Figure 1. From Figure 1, we can see that a given pair of support sample $I_{s}$ and query sample $I_{q}$, their feature representations $Z_{s}$ and $Z_{q}$ are extracted through ResNet 12. Henceforth, *Z* will represent both $Z_{s}$ and $Z_{q}$. These features exist in the space $R^{C\times H\times W}$, where *C* denotes the number of channels, *H* the height, and *W* the width.

Subsequently, the ESCA module enhances these base features through self-enhanced correlational attention, resulting in refined features $F_{s}$ and $F_{q}$. The output of the ESCA module directly serves as the input to the MBJ module, creating a coherent processing pipeline. This ensures that the enhancements made by the ESCA module are fully utilized by the MBJ module, facilitating more precise similarity computations.

The MBJ module uses these enhanced features to construct a reliable similarity matrix with Cross-Correlational Attention (CCA) and Cross Softmax Attention (CSA) techniques. The MBJ module further refines feature representation based on the ESCA output. The synergy between these two modules results in more accurate and robust final feature representations, providing higher quality inputs for the classification task.

This cascaded structure, from ESCA to MBJ, not only enhances the expressive capacity of features but also improves the model’s understanding of inter-sample relationships, ultimately boosting classification performance.

### 3.2. Enhanced Self-Correlation Attention

**Figure 2:**
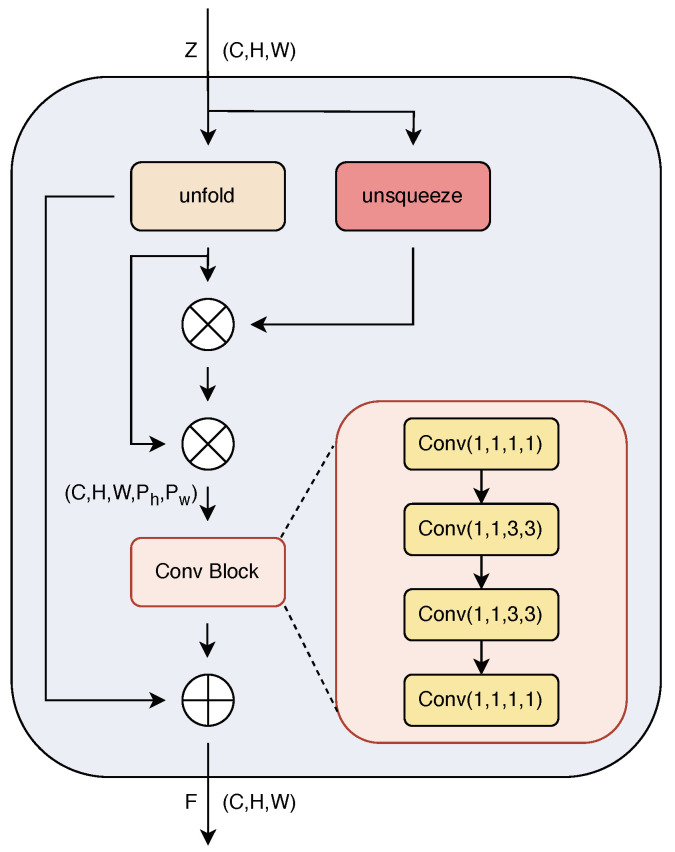
The architecture of ESCA module.

This subsection focuses on the structure of ESCA module, which is shown in Figure 2. From Figure 2, we can observe that the purpose of the ESCA module is to extract more prominent local region features from the base image features Z extracted via ResNet 12, unveiling deeper structures and relationships within the feature maps for self-attention enhancement, thereby providing reliable inputs for the MBJ.

The dimension of *Z* is C×H×W. Initially, an unfold operation is performed on *Z*, transforming the feature map *Z* into a series of blocks containing information of local regions, and reshaping these blocks into tensors with specified kernel sizes. Subsequently, these tensors undergo Hadamard product operations with the original feature map *Z* to obtain preliminary local self-correlational features *L*. The implementation of *L* can be specifically represented as Equation (1). Upon obtaining *L*, a second Hadamard product operation is conducted between *L* and the unfolded tensor to further obtain Enhanced Self-Correlational features *E*. The specific implementation of *E* can be represented as Equation (2).

(1) $$L=\frac{Z(x)}{\left||Z(x)|\right|}⊙\frac{Z(x+o)}{\left||Z(x+o)|\right|}$$

(2) $$E=L\;⊙\;\frac{Z(x+o)}{\left||Z(x+o)|\right|}$$

In the process of extracting self-correlational features, we apply element-wise multiplication (Hadamard product) to directly compute the preliminary correlation *L* among local features. This step reveals the interactions between each feature point and the features within its immediate neighborhood. Subsequently, a second element-wise multiplication is performed, which not only further strengthens the computed self-correlational properties but also deepens the correlational features. This operation re-weights the initially formed self-correlational mapping. The purpose of this is to enhance and refine the self-correlational features, capturing richer contextual information and the complex inter-dependencies between features. Mathematically, this is equivalent to self-correcting the self-correlational tensor, making it more sensitive to subtle variations in the original input, thereby providing a highly distinctive feature representation for subsequent feature analysis or classification tasks.

Within the framework of our work, we refined the feature extraction mechanism for the Enhanced Self-Correlational feature *E* by introducing a convolutional module. At the initial stage of processing, we employed a two-dimensional convolutional layer to reduce the number of feature map channels, effectively alleviating the computational pressure on the model while retaining key feature information. Subsequently, we deployed two three-dimensional convolutional layers, which fine-tune the spatial resolution of features along the $K_{h}\times K_{w}$ dimensions of *E*, aiming to compress the feature dimensions from $C\times H\times W\times K_{h}\times K_{w}$ to C×H×W×1×1. After this series of operations, we restored the channel dimension of features through an adjoining two-dimensional convolutional layer, ensuring that the produced self-correlational feature representation is consistent in spatial dimensions with the original feature *Z*. The design of the entire convolutional module is intended to enhance the network’s capability in capturing image details and complex structures, thereby deepening the model’s comprehensive understanding of the depth representation information of image content. Since the self-correlational feature representation and the original feature *Z* are consistent in spatial dimensions, combining the two features during the analysis provides the model with a more comprehensive understanding of features.

Thus, by combining them, we obtain the Enhanced Self-Correlational feature *F* after self-attention:

(3) F=ConvBlock(E)+Z

### 3.3. Multi-Branch Joint Module

**Figure 3:**
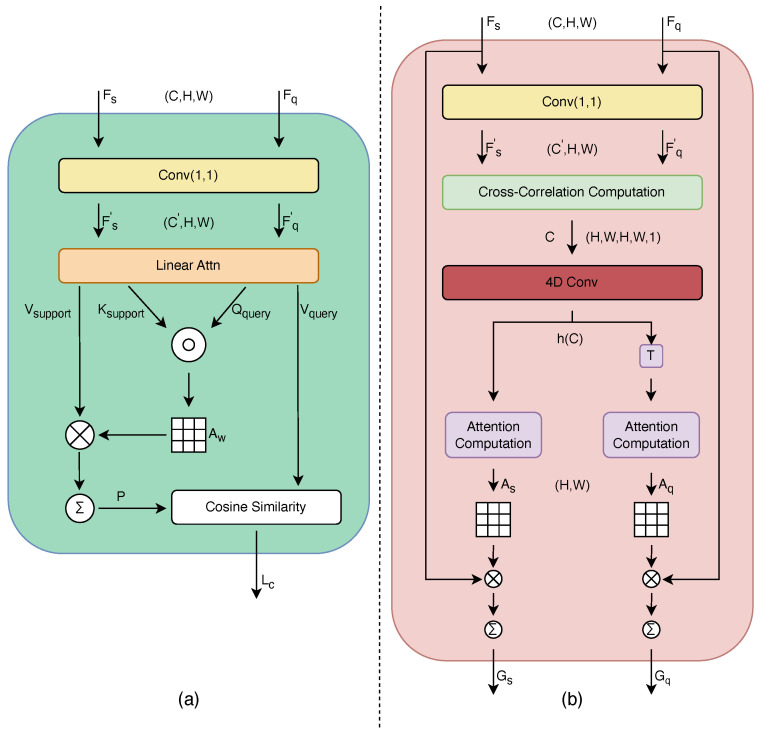
The architecture of the MBJ module. (**a**) CSA module and (**b**) CCA module.

In this subsection, we will explore the structure of MBJ module which is shown in Figure 3. Figure 3 provides a visual representation, from which we can deduce that MBJ takes the ESCA-processed support sample features $F_{s}$ and query sample features $F_{q}$ as inputs.This module employs two complementary attention mechanisms: Cross-Correlational Attention (CCA) and Cross Softmax Attention (CSA), each operating on a separate path. The rationale behind choosing these two techniques lies in their ability to capture different aspects of feature relationships, thereby providing a more comprehensive and accurate feature representation for classification tasks.

CCA is selected for its capacity to evaluate spatial relationships between images through geometric matching. By computing cross-correlations and applying 4D convolutional matching, CCA effectively captures local structural similarities between support and query samples. This is particularly beneficial in scenarios where spatial arrangements of features are crucial for classification.

On the other hand, CSA is employed for its strength in modeling global contextual information. By utilizing a softmax-based attention mechanism, CSA can identify and emphasize the most relevant features across the entire feature space. This global perspective complements the local focus of CCA, allowing the model to consider both fine-grained details and overarching patterns in the classification process.

The combination of these two attention mechanisms enables MBJ to generate a pair of embedding vectors that encapsulate both local and global feature relationships. Subsequently, a pairwise cosine similarity calculation is conducted for a detailed analysis of these features. Finally, the results of the similarity calculations are aggregated to form a comprehensive feature similarity assessment, leveraging the strengths of both CCA and CSA for improved classification accuracy.

#### 3.3.1. Cross-Correlational Attention

On the first support path, the ESCA outputs $F_{s}$ and $F_{q}$ are taken as inputs. Both inputs are first passed through a 1×1 convolutional layer to reduce the number of channels and extract important information, obtaining refined features $F_{s}^{\prime }$ and $F_{q}^{\prime }$. Subsequently, their cross-correlation is computed to evaluate and quantify the similarity between features, with the specific calculation as follows:

(4) $$C=sim(F_{s}^{\prime }\;,\;F_{q}^{\prime })$$

where sim represents the cosine similarity. Upon obtaining *C*, its reliability is enhanced through 4D convolutional matching, which involves two stages of 4D convolution: the first stage employs multiple matching kernels to expand the channels of the correlation tensor, while the second stage aggregates these tensors to form a single, more accurate 4D correlation tensor. Additionally, batch normalization and ReLU activation function are utilized to improve feature representation, thereby facilitating a better understanding of spatial relationships between images during geometric matching.

After the convolutional matching, we can construct the cross-attention between the support and query images. The calculation formula for the cross-attention map $A_{s}$ of the support image is as follows:

(5) $$A_{s}\left(x_{s}\right)=\frac{1}{HW}\sum_{x_{q}}\frac{\exp\left(h\left(x_{s},x_{q}\right)/\gamma \right)}{\sum_{x_{s}^{\prime }}\exp\left(h\left(x_{s}^{\prime },x_{q}\right)/\gamma \right)}$$

In the computation of attention, γ acts as the temperature coefficient, controlling the sensitivity of the matching scores, where *x* denotes specific positions within the feature maps. $h(x_{s},x_{q})$ represents the matching score between a position $x_{s}$ in the support and a position $x_{q}$ in the query. Thus, the expression for the average matching probability between a specific position on the support image and the corresponding position on the query image is denoted as $A_{s}$. The cross-attention for the query image, $A_{q}$, can be calculated in a similar manner. Upon obtaining $A_{s}$ and $A_{q}$, these attentions are fused with the original features $F_{s}$ and $F_{q}$ to yield the joint cross-attention maps $G_{s}$ and $G_{q}$.

Finally, the cosine similarity between $G_{s}$ and $G_{q}$ is computed, resulting in $L_{a}$, which is specifically represented as follows:

(6) $$\begin{array}{cc} L_{a} & =\frac{G_{s}\cdot G_{q}}{∥G_{s}∥∥G_{q}∥}\\ \; & =\frac{\left[\sum_{x_{s}}A_{s}\left(x_{s}\right)F_{s}\right]\cdot \left[\sum_{x_{q}}A_{q}\left(x_{q}\right)F_{q}\right]}{∥\sum_{x_{s}}A_{s}\left(x_{s}\right)F_{s}∥∥\sum_{x_{q}}A_{q}\left(x_{q}\right)F_{q}∥} \end{array}$$

#### 3.3.2. Cross Softmax Attention

In another branch, the process begins with a point convolution layer to acquire refined features $F_{s}^{\prime }$ and $F_{q}^{\prime }$. Subsequently, $F_{s}^{\prime }$ and $F_{q}^{\prime }$ are reorganized into new dimensions to preserve the dimensions of the number of classes and the number of queries during the computation process. The new representations $V_{support}$, $K_{support}$, $Q_{query}$, and $V_{query}$ are obtained through linear layers, transformed by the weight matrices $\theta_{q}$, $\theta_{k}$, and $\theta_{v}$. The specific representations are as follows:

(7) $$\left\{\begin{array}{c} V_{support}=F_{s}^{\prime }\cdot \theta_{v}\\ V_{query}=F_{q}^{\prime }\cdot \theta_{v}\\ K_{support}=F_{s}^{\prime }\cdot \theta_{k}\\ Q_{query}=F_{q}^{\prime }\cdot \theta_{q} \end{array}\right.$$

To further elaborate, we compute the dot product similarity between $Q_{query}$ and $K_{support}$, followed by a scaling operation through division by $\sqrt{d}$, where *d* represents the dimensionality of the new representations. This scaling operation aids in mitigating potential gradient instability. Subsequently, the softmax function is applied to precisely obtain the attention distribution matrix $A_{w}$. The output of the softmax function is a probability distribution, converting larger dot product values into higher probabilities, and smaller values into lower probabilities. This distribution reflects the importance of each query feature relative to each prototype feature. The attention distribution matrix $A_{w}$ is then utilized to weight $V_{support}$ to generate the weighted output *P*. This representation captures the most significant relationships between query and support features, assigning higher weight in determining the final class decision. Through this mechanism, the attention allows the model to focus on the most informative features, thereby enhancing its accuracy and generalizability.

Finally, cosine similarity is computed once more between the attention-weighted feature representation *P* and $V_{query}$, culminating in $L_{b}$. This step further quantifies the alignment between the query features and the attention-weighted features. The specific representation is as follows:

(8) $$\begin{array}{cc} L_{b} & =\frac{P\cdot V_{query}}{∥P∥\;∥V_{query}∥}\\ \; & =\frac{\left(A_{w}\cdot V_{support}\right)\cdot V_{query}}{∥A_{w}\cdot V_{support}∥\;∥V_{query}∥} \end{array}$$

(9) $$\begin{array}{c} A_{w}=softmax\left(\left(q_{query}\cdot K_{support}\right)/\sqrt{d}\right) \end{array}$$

## 4. Training Strategy of EMNet

### 4.1. Multi-Branch Strategy

In the training strategy of EMNet, we employ two logical values, $L_{a}$ and $L_{b}$, as crucial parts of the model’s output, to guide the learning path of the model. To precisely balance the contributions of these two logical values within the model, we introduce two hyperparameters, α and β. The specific representation is as follows:

(10) $$Logit=\alpha L_{a}+\beta L_{b}$$

The logical value $L_{a}$ reflects the consistency between the support and query images after processing through the attention mechanism. Its calculation is based on the hypothesis that if a query image is highly consistent with images in the support set, they should belong to the same category. Thus, $L_{a}$ enhances the model’s sensitivity to those features crucial for category discrimination, thereby improving classification accuracy.

On the other hand, the logical value $L_{b}$ further deepens this measure of consistency. It considers not only the similarity of basic features but also the complex relationships between features learned through the model. $L_{b}$ aims to capture a more detailed correspondence between query features and weighted features, thereby providing richer information to guide the model’s judgment.

Through precise adjustment of α and β, we can control the weight of $L_{a}$ and $L_{b}$ in the final decision, thus endowing the model with adaptability and generalization capability in complex data environments. This approach allows us to maintain the model’s sensitivity to new samples while ensuring it is not misled by irrelevant features, which is particularly important in the context of few-shot learning.

### 4.2. Loss Function

The anchor classification loss, $L_{anchor}$, is computed by introducing a fully connected classification layer after obtaining the model’s average pooling base representation $z_{q}$. This method aims to guide the model in accurately classifying query samples within the training category set $C_{train}$. Through this loss function, we expect the model to enhance its sensitivity to different category features, ensuring accurate identification of each category on the training set.

(11) $$L_{anchor}=-log\frac{exp\left(w_{c}^{T}z_{q}+b_{c}\right)}{\sum_{c^{\prime }=1}^{|C_{train}|}exp\left(w_{c^{\prime }}^{T}z_{q}+b_{c^{\prime }}\right)}$$

The relevance metric classification loss, $L_{metric}$, aims to compute the similarity between query sample embeddings and support sample prototype embeddings using the cosine similarity measure. Before calculating this loss, the model averages the embedding vectors of query and support samples for each category to form prototype embeddings. The purpose of this loss is to motivate the model to map query sample embeddings onto the nearest prototype embedding of the same class. Here, γ is a temperature factor used to adjust the similarity calculation.

(12) $$\begin{array}{c} L_{metric}=-log\left[\frac{exp\left(L_{a}^{\left(n\right)}/\gamma \right)}{\sum_{n^{\prime }=1}^{N}exp\left(L_{a}^{\left(n^{\prime }\right)}/\gamma \right)}\right]\\ -log\left[\frac{exp\left(L_{b}^{\left(n\right)}/\gamma \right)}{\sum_{n^{\prime }=1}^{N}exp\left(L_{b}^{\left(n^{\prime }\right)}/\gamma \right)}\right] \end{array}$$

The final loss function combines these two losses, expressed as $\mathrm{Loss}=\rho L_{anchor}+\lambda L_{metric}$. Here, ρ and λ are hyperparameters used to balance the weights of the two losses. It is important to note that, during model inference, the fully connected layer used for calculating $L_{anchor}$ will be disregarded, making the model’s predictions based on the similarity between the query sample and the nearest prototype.

## 5. Experiments

### 5.1. Datasets

We conducted a series of experiments on three significant and widely recognized few-shot classification benchmark datasets to train and evaluate our method. These datasets include miniImageNet [11], CIFAR-FS [29], and CUB-200-2011 [30]. To further explore the effects of key components in our method, ablation studies were also performed on subsets of these three datasets. The miniImageNet dataset, derived from the ImageNet dataset [31], consists of 100 categories randomly divided into 64 base classes, 16 validation classes, and 20 novel classes, with each category containing 600 images. The CIFAR-FS dataset originates from CIFAR100 [32], featuring 100 categories also randomly allocated into 64 base classes, 16 validation classes, and 20 novel classes, with each class containing 600 images. The CUB-200-2011 (CUB) dataset focuses on fine-grained bird species classification, comprising 100/50/50 categories for training, validation, and testing, respectively. The choice of the miniImageNet, CUB-200-2011, and CIFAR-FS datasets for our experiments is primarily to ensure comprehensive, diverse, and fair evaluations. These datasets are representative in the field of few-shot learning and allow for a thorough assessment of the model’s performance across different scenarios. By comparing our results with other methods, we can validate the effectiveness and superiority of the proposed EMNet model.

### 5.2. Experimental Details

We selected ResNet 12 as the backbone network, which accepts image inputs of size 84×84 pixels and produces a high-dimensional feature representation of 5×5×640. Our method was implemented using the PyTorch 1.7.1 framework and trained on an NVIDIA RTX 3060 graphics processor with 12GB of VRAM.The NVIDIA RTX 30 series display chip is manufactured using an 8nm process, features the GA106-300/302 core, has a base clock speed of 1320MHz and a boost clock speed of 1780MHz, and includes 3584 CUDA cores. For all experiments, a unified batch size of 64 and γ=0.2 were adopted. For the five-way one-shot experiments, a maximum of 100 training epochs was consistently used, while for the five-way five-shot experiments, 80 epochs were set as the maximum. For the CUB-200-2011, CIFAR-FS, and miniImageNet datasets, identical values of λ and ρ were used, being 1.25, 0.35, and 0.1, respectively. The α values were set to 1.25, 1.25, and 2.25, respectively, with a unified β=0.25. Learning rates were individually set to 0.1, 0.05, and 0.1 for each dataset, respectively.

### 5.3. Performance of EMNet

In this part, we will provide performance of EMNet. Looking at Table 1, Table 2 and Table 3, we can see that in our research, through experiments on three authoritative benchmark datasets, we meticulously compared the performance of EMNet with existing methods. In the mentioned N-way K-shot scenario, the support set consists of n distinct categories, with each category providing k samples. The experimental results were encouraging: EMNet outperformed previous methods across all three datasets. This empirical study solidly demonstrates EMNet’s superior capability in few-shot image classification tasks.

The choice of a backbone network different from ResNet 12 is due to the fact that the cited works in their articles also chose backbone networks different from ResNet 12, but lack experimental data with ResNet 12 as the backbone network. Therefore, when citing the experimental results of these articles, there are no data with ResNet 12 as the backbone network.

It is well known that larger backbone networks contain more layers and parameters, implying that the model can learn more complex and diverse features, theoretically possessing stronger representation capabilities. However, looking at the experimental results, models that chose other larger backbone networks (such as ResNet 18, ResNet 34, WRN-28-10, etc.) actually performed worse than EMNet with ResNet 12. This indirectly demonstrates that EMNet is more efficient in feature extraction and utilization, able to extract more representative features even with a smaller backbone network. The number of parameters in the ESCA module is approximately 0.6 MB, while the number of parameters in the MBJ module is around 0.94 MB. The total number of parameters in EMNet is approximately 1.54 MB.

**Figure 4:**
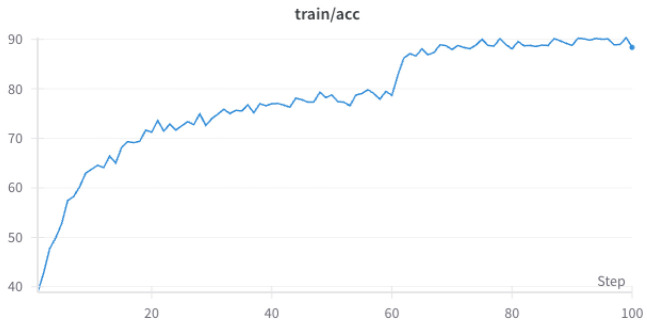
CUB-200-2011 EMNet training accuracy.

**Figure 5:**
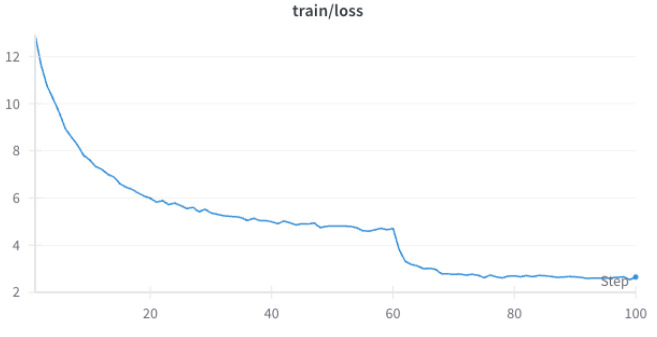
CUB-200-2011 EMNet training loss.

Here, we will describe the training data of our proposed EMNet which is training on CUB-200-2011, the detailed result is shown in Figure 4 and Figure 5. From Figure 4 and Figure 5, it can be seen that the training accuracy of EMNet steadily increases with the number of training epochs. After 60 epochs, the rate of improvement begins to slow down, and during the 90–100 epochs, the accuracy stabilizes. On the other hand, the training loss decreases with the number of training epochs, indicating that the model is continuously optimizing and reducing prediction errors.

### 5.4. Ablation Experiments

To validate the effectiveness of our proposed method, we conducted a series of ablation studies focusing on the impact of the ESCA and MBJ modules. The results of these studies are documented in Table 4. When only the ESCA module was activated, its output was used as the input for the CCA module in RENet; conversely, when only the MBJ module was operational, the output of SCR in RENet served as the input for the MBJ module, culminating in the final results for classification.

The outcomes of the ablation experiments highlighted that the ESCA module exhibited an advantage in providing richer feature representations, while the MBJ module enhanced the model’s feature matching and alignment capabilities, which is particularly crucial in scenarios with limited sample sizes. When combined, they complement each other, offering a more comprehensive improvement in the model’s performance on few-shot learning tasks.

### 5.5. Attention Comparison

**Figure 6:**
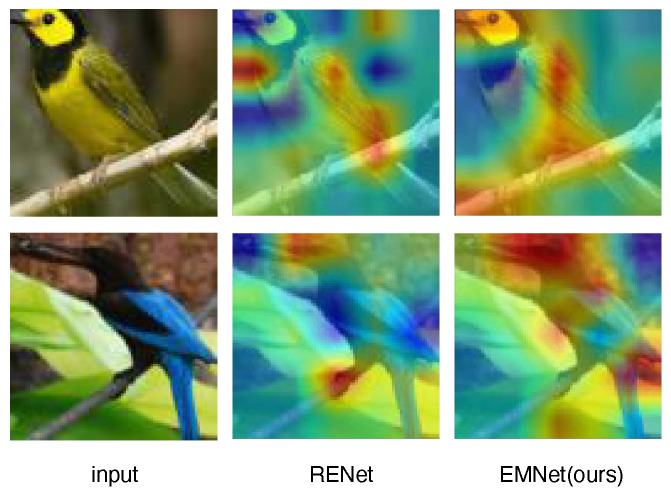
Attention comparison with RENet [7].

The attentional effects result of EMNet is shown in Figure 6. In comparison to RENet, EMNet demonstrates higher performance in generating attention maps. EMNet exhibits more focused attention on crucial areas, such as the bird’s head and back, with higher activation values. This allows for more accurate capture of essential image features. EMNet also shows improved detail in feature extraction, with clearer and more intense activation in key regions.

Conversely, RENet’s attention maps are more diffuse, potentially leading to interference from non-critical areas. Its activation values are less distinct, indicating less precise feature extraction.

EMNet’s higher attention concentration and more localized distribution of activation values suggest enhanced focus on critical features, potentially improving classification and recognition accuracy. Overall, EMNet outperforms RENet in attention focus, feature detail capture, and emphasis on key areas, indicating its superior efficacy in visual recognition tasks.

## 6. Conclusions

In this work, we introduce the ESCA mechanism and the MBJ module. Utilizing the self-attention framework, ESCA is capable of highlighting significant feature regions and reinforcing their representations to better capture both the structural and semantic information in images. The core of this mechanism lies in its ability to not only identify salient features within an image but also to ensure that these features receive greater weight and attention during the model’s decision-making process through self-attention.

The MBJ module, with its multi-branch architecture, effectively filters out task-irrelevant features while promoting the in-depth exploration of shared features across images. It leverages the interaction between intra-class similarity and inter-class variability, resulting in a composite attention pattern that focuses on multiple critical regions within the image. The purpose of this layer is to synthesize the learning outcomes from each branch, distilling features that can represent the core attributes of each category, thereby enhancing the model’s ability to capture fine details.

In our experiments, MBJ demonstrated its effectiveness in constructing highly discriminative composite feature representations, which are crucial for improving the accuracy of the model when handling few-shot image classification tasks. Experimental validation on standard datasets highlighted the MBJ’s significant advantages in boosting model generalization capability and refining classification granularity.

Our experimental results indicate that the EMNet model, comprising the ESCA module and the MBJ block, performs robustly through end-to-end training across three public datasets. Furthermore, ablation studies confirmed the efficacy of the model components.

Although EMNet demonstrates excellent performance in feature extraction and generalization, its multi-branch structure and attention mechanisms increase computational complexity, especially when dealing with high-resolution images or large datasets, leading to higher computational resource requirements. It is possible to consider introducing more complex attention mechanisms, such as multi-head attention or variants of self-attention, to further enhance the model’s feature extraction and generalization capabilities. In medical image analysis, where data annotation is costly and samples are scarce, EMNet can be utilized for tasks such as medical image classification and lesion detection, assisting doctors in making more accurate diagnoses.

## Figures and Tables

**Table 1:** Performance of EMNet on the CUB-200-2011 dataset. Results are reported as mean accuracy (%) ± standard deviation.

Method	Backbone	Five-Way One-Shot	Five-Way Five-Shot
ProtoNet [12]	ResNet 12	66.09±0.92	82.50±0.58
RelationNet [13]	ResNet 34	66.20±0.99	82.30±0.58
MAML [14]	ResNet 34	67.28±1.08	83.47±0.59
cosine classifier [18]	ResNet 12	67.30±0.86	84.75±0.60
MatchNet [11]	ResNet 12	71.87±0.85	85.08±0.57
NegMargin [33]	ResNet 18	72.66±0.85	89.40±0.43
FEAT [5]	ResNet 12	73.27±0.22	85.77±0.14
DeepEMD [34]	ResNet 12	75.65±0.83	88.69±0.50
RENet [7]	ResNet 12	79.49±0.44	91.11±0.24
**EMNet (ours)**	ResNet 12	80.76±0.43	91.65±0.32

**Table 2:** Performance of EMNet on the miniImageNet dataset. Results are reported as mean accuracy (%) ± standard deviation.

Method	Backbone	Five-Way One-Shot	Five-Way Five-Shot
ProtoNet [12]	ResNet 12	62.39±0.21	80.53±0.14
MetaOptNet [35]	ResNet 12	62.64±0.82	78.63±0.46
MatchNet [11]	ResNet 12	63.08±0.80	75.99±0.60
CAN [36]	ResNet 12	63.85±0.48	79.44±0.34
NegMargin [33]	ResNet 12	63.85±0.81	81.57±0.56
CTM [37]	ResNet 18	64.12±0.82	80.51±0.13
DeepEMD [34]	ResNet 12	65.91±0.82	82.41±0.56
FEAT [5]	ResNet 12	66.78±0.20	82.05±0.30
RENet [7]	ResNet 12	67.60±0.44	82.58±0.30
**EMNet (ours)**	ResNet 12	67.62±0.43	83.03±0.30

**Table 3:** Performance of EMNet on the CIFAR-FS dataset. Results are reported as mean accuracy (%) ± standard deviation.

Method	Backbone	Five-Way One-Shot	Five-Way Five-Shot
RFS-simple [38]	ResNet 12	71.5±0.80	86.0±0.5
ProtoNet [12]	ResNet 12	72.2±0.70	83.5±0.5
MetaOptNet [35]	ResNet 12	72.6±0.7	84.3±0.5
Boosting [39]	WRN-28-10	73.6±0.3	86.6±0.32
RENet [7]	ResNet 12	74.51±0.46	86.60±0.32
MTNet [40]	ResNet 12	75.19±0.42	86.66±0.34
**EMNet (ours)**	ResNet 12	75.38±0.46	86.84±0.32

**Table 4:** Results of EMNet ablation experiments on three publicly available datasets.

ESCA	MBJ	miniImageNet		CUB-200-2011		CIFAR-FS	
		One-Shot	Five-Shot	One-Shot	Five-Shot	One-Shot	Five-Shot
✓	×	67.60	82.80	79.83	91.48	74.64	86.76
×	✓	67.59	82.80	80.38	91.47	75.09	86.65
✓	✓	**67.62**	**83.03**	**80.76**	**91.65**	**75.38**	**86.84**

## Data Availability

The original contributions presented in the study are included in the articlel, further inquiries can be directed to the corresponding authors.
